# Higher Hand Grip Strength Is Associated With Greater Radius Bone Size and Strength in Older Men and Women: The Framingham Osteoporosis Study

**DOI:** 10.1002/jbm4.10485

**Published:** 2021-03-30

**Authors:** Robert R. McLean, Elizabeth J. Samelson, Amanda L. Lorbergs, Kerry E. Broe, Marian T. Hannan, Steven K. Boyd, Mary L. Bouxsein, Douglas P. Kiel

**Affiliations:** ^1^ Hinda and Arthur Marcus Institute for Aging Research, Hebrew SeniorLife Boston Massachusetts USA; ^2^ CorEvitas, LLC Waltham Massachusetts USA; ^3^ Department of Medicine Beth Israel Deaconess Medical Center and Harvard Medical School Boston Massachusetts USA; ^4^ Canadian Frailty Network Kingston Ontario Canada; ^5^ McCaig Institute for Bone and Joint Health, Cumming School of Medicine, University of Calgary Calgary Alberta Canada; ^6^ Center for Advanced Orthopaedic Studies, Beth Israel Deaconess Medical Center Boston Massachusetts USA; ^7^ Department of Orthopedic Surgery Harvard Medical School Boston Massachusetts USA

**Keywords:** AGING, BONE‐MUSCLE INTERACTIONS, MUSCLE STRENGTH, HR‐pQCT, EPIDEMIOLOGY

## Abstract

Mechanical loading by muscles elicits anabolic responses from bone, thus age‐related declines in muscle strength may contribute to bone fragility in older adults. We used high‐resolution peripheral quantitative computed tomography (HR‐pQCT) to determine the association between grip strength and distal radius bone density, size, morphology, and microarchitecture, as well as bone strength estimated by micro–finite element analysis (μFEA), among older men and women. Participants included 508 men and 651 women participating in the Framingham Offspring Study with grip strength measured in 2011–2014 and HR‐pQCT scanning in 2012–2015. Separately for men and women, analysis of covariance was used to compare HR‐pQCT measures among grip strength quartiles and to test for linear trends, adjusting for age, height, weight, smoking, and physical activity. Mean age was 70 years (range, 50–95 years), and men had higher mean grip strength than the women (37 kg vs. 21 kg). Bone strength estimated by μFEA‐calculated failure load was higher with greater grip strength in both men (*p* < 0.01) and women (*p* = 0.04). Higher grip strength was associated with larger cross‐sectional area in both men and women (*p* < 0.01), with differences in area of 6% and 11% between the lowest to highest grip strength quartiles in men and women, respectively. Cortical thickness was positively associated with grip strength among men only (*p* = 0.03). Grip strength was not associated with volumetric BMD (vBMD) in men. Conversely, there was a trend for lower total vBMD with higher grip strength among women (*p* = 0.02), though pairwise comparisons did not reveal any statistically significant differences in total vBMD among grip strength quartiles. Bone microarchitecture (cortical porosity, trabecular thickness, trabecular number) was not associated with grip strength in either men or women. Our findings suggest that the positive association between hand grip strength and distal radius bone strength may be driven primarily by bone size. © 2021 The Authors. *JBMR Plus* published by Wiley Periodicals LLC on behalf of American Society for Bone and Mineral Research.

## Introduction

Mechanical loading elicits anabolic responses from bone,^(^ ^1^ ^)^ with skeletal muscles generating up to 70% of the physiological forces applied to bone.^(^ ^2^ ^)^ Accordingly, epidemiologic studies consistently demonstrate that greater muscle strength is directly associated with bone mass, density and size across the life span.^(^ ^3^ ^)^ Starting in the fifth decade of life, muscle strength is steadily lost with aging, which parallels the concomitant loss of bone mass.^(^ ^4^ ^)^ Thus, decline in muscle strength may be an important contributor to age‐related bone loss and bone fragility in older adults.

The distal radius is a non‐weight‐bearing site where the attached muscles exert forces on the bone, and some of these forces^(^ ^5^, ^6^ ^)^ are involved in the generation of hand grip strength. Several studies utilizing dual‐energy x‐ray absorptiometry (DXA) and peripheral quantitative computed tomography (pQCT) to assess bone in older adults have established that lower grip strength is associated with lower bone mass, density, and size at the distal radius,^(^ ^7^, ^8^, ^9^, ^10^, ^11^, ^12^, ^13^, ^14^, ^15^ ^)^ yet these technologies have some important limitations. Areal bone mineral density (aBMD) measured by DXA is a two‐dimensional imaging method that cannot measure volumetric bone mineral density (vBMD) or separate cortical and trabecular compartments. Although pQCT assesses cortical and trabecular vBMD, bone size, and geometry‐based estimates of bone strength, it does not capture microarchitecture, an important determinant of bone strength. Thus, our understanding of the contribution of muscle strength to bone strength in older adults is incomplete.

High‐resolution peripheral quantitative computed tomography (HR‐pQCT) overcomes the limitations of DXA and pQCT to facilitate a comprehensive examination of bone, including cortical and trabecular bone microarchitecture and bone strength estimated by micro–finite element analysis (μFEA). To date, a single study including men only has utilized HR‐pQCT to examine the association between grip strength and bone outcomes at the distal radius in older adults,^(^ ^15^ ^)^ yet evidence suggests that there are likely important sex differences in the muscle‐bone relation with aging.^(^ ^16^ ^)^ Furthermore, bone strength estimated by μFEA has not yet been examined in older adults. Addressing these important knowledge gaps will provide a better understanding of how muscle strength influences bone, which may inform new strategies for maintaining musculoskeletal health with aging.

Our objective was to determine the associations between grip strength and HR‐pQCT measures of distal radius bone density, size, morphology, and microarchitecture, and overall bone strength estimated by μFEA, among men and women aged 50 years and older. We hypothesized that greater grip strength would be associated with higher bone density and size, more favorable morphology and microarchitecture, and greater estimated bone strength.

## Subjects and Methods

### Participants

Study participants included members of the Framingham Study Offspring cohort. From 1971 to 1975, 5124 of the adult children, and their spouses, of the population‐based Framingham Original Cohort^(^ ^17^ ^)^ were enrolled (age range 5–70 years at enrollment) and subsequently examined at approximately 4‐year intervals to investigate familial risk factors for cardiovascular disease.^(^ ^18^ ^)^ The current study includes the 1159 Offspring cohort participants who were aged 50 years and older and had available data on grip strength ascertained at their primary Framingham visit in 2011–2014, and HR‐pQCT scans of sufficient quality (based on lack of movement artifacts) which were collected as part of call‐back visits for the Framingham Osteoporosis Study that were completed in 2012–2015. HR‐pQCT measures were collected an average of 1.8 years (range, 0–4.3 years) after grip strength was ascertained. Participants provided written informed consent, and the Institutional Review Board for Human Research at Boston University and Hebrew SeniorLife approved the study.

### HR‐pQCT

Bone density, size, morphology, and microarchitecture of the distal radius were measured in 2012–2015 using HR‐pQCT (XtremeCT; Scanco Medical AG, Bruttisellen, Switzerland), as described.^(^ ^19^, ^20^ ^)^ Briefly, scans of the nondominant forearm were acquired, unless the participant reported a history of fracture or metal in the scan region, in which case the contralateral forearm was scanned (*n* = 43). Using a scout view, the measurement region was defined by placing a reference line on the midpoint of the distal endplate of the radius. The scan region (9 mm in length) began 9.5 mm proximal to the reference line. Scans were reviewed for quality and graded on a five‐point movement artifact scale, with 5 being the most extreme movement.^(^ ^21^ ^)^ Scans graded 5 were excluded (<1% of all scans ascertained). Scans graded ≤4 were evaluated for volumetric density and size/morphology measures. For microarchitecture and μFEA measures, only those graded ≤3 were evaluated.

The SCANCO standard analysis (Image Processing Language, v6.0) was used to obtain total bone and trabecular compartment measures, whereas an extended cortical analysis algorithm^(^ ^22^ ^)^ calculated cortical bone measures. Bone parameters for the current analysis included density (total vBMD [mg/cm^3^], trabecular vBMD [mg/cm^3^], cortical vBMD [mg/cm^3^]), size/morphology (total cross‐sectional area [mm^2^], cortical area fraction [%], cortical thickness [mm]), and microarchitecture (cortical porosity [%], trabecular thickness [mm], trabecular number [mm^−1^]). Additionally, to estimate bone strength, failure load (N, Newtons) was calculated using linear μFEA based on axial compression conditions applied with 1% apparent strain, a tissue modulus of 6.829 GPa and Poisson's ratio of 0.3.^(^ ^23^ ^)^ All participants included in this analysis had complete information on all HR‐pQCT measures. At the time of the current analysis, scans from 17 participants had not yet completed μFEA analysis and are therefore missing failure load information. Precision estimates for HR‐pQCT measures have been reported.^(^ ^24^, ^25^ ^)^

### Grip strength

Grip strength (kg) was assessed during 2011–2014 using a JAMAR adjustable isometric hand‐held dynamometer (Model #5030J1; Sammons Preston/JLW Instruments, Chicago, IL, USA).^(^ ^26^ ^)^ Participants were seated in a chair with arms, forearms resting on the chair arms, elbows at approximately 90‐degree angles, and instructed to hold the dynamometer in the upright position with their wrist in a neutral position. They were asked to squeeze the dynamometer as hard as possible for 3 to 5 s, and three trials were attempted for each hand. For the current study, the maximum value from the hand of the forearm that was assessed for HR‐pQCT was used.

### Other variables

Covariate data were obtained at the same time as the grip strength assessment (2011–2014) and included sex, age (years), height (inches), weight (pounds), current smoking (yes/no), physical activity index score, and presence of type 2 diabetes. Height without shoes (inches) was measured to the nearest quarter inch with a stadiometer and, weight in light clothing without shoes (pounds) was measured with a standardized balance‐beam scale. Participants who reported smoking cigarettes regularly in the past year were considered current smokers. Physical activity was assessed using an index calculated as a weighted sum of the number of self‐reported hours spent on strenuous, moderate, and light activities, and at rest, for a typical day over the past year.^(^ ^27^, ^28^ ^)^ Type 2 diabetes was defined as fasting plasma glucose concentration >125 mg/dL or current use of insulin or oral hypoglycemic therapy.

### Statistical analyses

Due to differences in the distributions of grip strength and bone measures between women and men, analyses were conducted separately for men and women. To determine the association of grip strength with bone density, size, morphology, microarchitecture, and overall bone strength estimated by μFEA, analysis of covariance was used to calculate and compare least‐squares adjusted mean bone measures across quartiles of grip strength, with Tukey's adjustment for pairwise comparisons, and test for a linear trend across quartiles from lowest to highest. All analyses were adjusted for age, height, weight, smoking, and physical activity.

We recently showed that type 2 diabetes is associated with deficits in bone microarchitecture and smaller bone size.^(^ ^20^ ^)^ In addition, type 2 diabetes is associated with lower muscle strength,^(^ ^29^, ^30^ ^)^ thus the muscle strength–bone relation may be different in older adults with type 2 diabetes compared to those without diabetes. Accordingly, we conducted a sensitivity analysis, repeating analyses excluding participants with type 2 diabetes (men, *n* = 128; women, *n* = 90). Furthermore, original models were repeated considering diabetes as an additional covariate.

For all statistical hypothesis tests, a two‐sided *p* value <0.05 was considered statistically significant. Analyses were performed using PC‐SAS (version 9.4; SAS Institute, Cary, NC, USA).

## Results

Mean age of the 508 men and 651 women in our study was 70 years (range, 50–95 years), and all women were postmenopausal. There were numerical differences between the cohorts of men and women for several important characteristics. Compared to men, the women weighed less, were shorter, and had lower mean grip strength (Table 1). Women tended to have lower trabecular and total volumetric bone density, but similar cortical density, compared to men. Bone size and trabecular architecture were lower in women, though cortical porosity was greater in men. Finally, bone strength estimated by μFEA was higher in men than women.

**Table 1:** Characteristics of Framingham Osteoporosis Study participants (2012–2015) Who Had Grip Strength and Radius Bone Microarchitecture Measures on the Same Arm

Characteristic	Men (*n* = 508)	Women (*n* = 651)
Age (years), mean ± SD	70 ± 8	70 ± 7
Weight (pounds), mean ± SD	193 ± 32	158 ± 33
Height (inches), mean ± SD	68.57 ± 2.58	63.04 ± 2.49
Current smoker (%)	5.5	5.4
Framingham physical activity index, mean ± SD	36 ± 6	34 ± 5
Maximum grip strength (kg), mean ± SD	37 ± 9	21 ± 6
Trabecular density (mg/cm^3^), mean ± SD	187 ± 36	147 ± 39
Cortical density (mg/cm^3^), mean ± SD	953 ± 55	957 ± 62
Cortical thickness (mm), mean ± SD	0.97 ± 0.20	0.80 ± 0.19
Cortical porosity (%), mean ± SD	4.3 ± 1.7	3.7 ± 1.6
Cortical area fraction (%), mean ± SD	20.7 ± 5.1	20.4 ± 5.4
Total density (mg/cm^3^), mean ± SD	333 ± 63	295 ± 67
Total cross‐sectional area (mm^2^), mean ± SD	378 ± 61	253 ± 46
Trabecular thickness (mm), mean ± SD	0.070 ± 0.011	0.064 ± 0.011
Trabecular number (mm^−1^), mean ± SD	2.23 ± 0.26	1.91 ± 0.39
Failure load (N), mean ± SD^a^	3243 ± 577	1966 ± 378

Abbreviation: SD, standard deviation.

^a^ Missing: men, *n* = 9; women, *n* = 8.

### Bone density

Although volumetric bone density was not statistically significantly associated with grip strength in men (Table 2), women had lower volumetric bone density with greater grip strength (Table 3), and there was a statistically significant decreasing linear trend for total density (*p* trend = 0.02). Similar negative linear trends were observed for cortical and total density but did not reach statistical significance.

### Bone size and morphology

There were statistically significant positive associations of grip strength with cross‐sectional area for both men (*p* trend <0.01) and women (*p* trend <0.01). The highest grip strength quartiles for men and women had 6% (*p* < 0.01) and 11% (*p* < 0.01) greater area, respectively, compared to the lowest quartiles (Tables 2 and 3). Grip strength in men was not associated with cortical area fraction (*p* trend = 0.49). In women there was a statistically significant trend (*p* trend = 0.02) for decreasing cortical area fraction with greater grip strength, though the magnitude of the association was modest, with 0.4% difference in means between the lowest and highest grip strength quartiles. Cortical thickness was higher with greater grip strength in men (*p* trend = 0.03), with the adjusted mean 5% greater in the highest versus the lowest quartile (*p* = 0.04), but there was no statistically significant association among women (*p* trend = 0.21).

**Table 2:** Least Squares‐Adjusted Mean Radius Bone Parameters by Quartiles of Maximum Grip Strength Among 508 Men in the Framingham Osteoporosis Study

	Grip strength quartile				
Bone parameter	Q1 (*n* = 123)	Q2 (*n* = 139)	Q3 (*n* = 120)	Q4 (*n* = 124)	*p* trend
Density, mean ± SE					
Total density (mg/cm^3^)	330 ± 6	328 ± 5	335 ± 6	339 ± 6	0.24
Trabecular density (mg/cm^3^)	185 ± 3	185 ± 3	187 ± 3	190 ± 4	0.34
Cortical density (mg/cm^3^)	950 ± 5	949 ± 4	953 ± 5	960 ± 5	0.18
Size/morphology, mean ± SE					
Total area (mm^2^)	367.9 ± 5.1	371.4 ± 4.6	382.1 ± 4.9	390.8 ± 5.2* ^,^ **	**<0.01**
Cortical area fraction (%)	20.6 ± 0.5	20.5 ± 0.4	21.0 ± 0.5	21.0 ± 0.5	0.49
Cortical thickness (mm)	0.948 ± 0.019	0.946 ± 0.017	0.987 ± 0.018	0.999 ± 0.019*	**0.03**
Microarchitecture, mean ± SE					
Cortical porosity (%)	4.2 ± 0.2	4.2 ± 0.1	4.4 ± 0.1	4.2 ± 0.2	>0.99
Trabecular thickness (mm)	0.070 ± 0.001	0.070 ± 0.001	0.069 ± 0.001	0.070 ± 0.001	0.89
Trabecular number (mm^−1^)	2.20 ± 0.02	2.21 ± 0.02	2.26 ± 0.02	2.26 ± 0.03	0.06
Strength, mean ± SE					
Failure load (N)	3101.6 ± 51.8	3147.0 ± 45.5	3303.0 ± 49.3* ^,^ **	3430.8 ± 52.3* ^,^ **	**<0.01**

*Note*: Bold values are significant at *p* < 0.05. Least squares values are adjusted for age, weight, height, physical activity, smoking.

Abbreviation: Q, quartile; SE, standard error.

* *p* < 0.05 versus Q1.

** *p* < 0.05 versus Q2.

### Bone microarchitecture

For both men and women, grip strength was not associated with either cortical porosity or trabecular thickness (Tables 2 and 3). Similarly, there was no association with trabecular number in women. Although mean trabecular number tended to be higher across quartiles of ascending grip strength quartile in men, the linear trend was not statistically significant (*p* trend = 0.06).

**Table 3:** Least Squares‐Adjusted Mean Radius Bone Parameters by Quartiles of Maximum Grip Strength Among 651 Women in the Framingham Osteoporosis Study

	Grip strength quartile				
Bone parameter	Q1 (*n* = 160)	Q2 (*n* = 159)	Q3 (*n* = 168)	Q4 (*n* = 164)	*p* trend
Density, mean ± SE					
Total density (mg/cm^3^)	304 ± 5	297 ± 5	294 ± 5	287 ± 5	**0.02**
Trabecular density (mg/cm^3^)	149 ± 3	147 ± 3	147 ± 3	144 ± 3	0.35
Cortical density (mg/cm^3^)	963 ± 5	958 ± 4	956 ± 4	950 ± 5	0.08
Size/morphology, mean ± SE					
Total area (mm^2^)	241.5 ± 3.3	248.3 ± 3.0	254.6 ± 2.9^*^	267.1 ± 3.2* ^,^ **	**<0.01**
Cortical area fraction (%)	21.1 ± 0.4	20.4 ± 0.4	20.2 ± 0.4	19.7 ± 0.4	**0.02**
Cortical thickness (mm)	0.813 ± 0.015	0.795 ± 0.014	0.793 ± 0.013	0.785 ± 0.014	0.21
Microarchitecture, mean ± SE					
Cortical porosity (%)	3.6 ± 0.1	3.8 ± 0.1	3.7 ± 0.1	3.8 ± 0.1	0.57
Trabecular thickness (mm)	0.065 ± 0.001	0.064 ± 0.001	0.063 ± 0.001	0.063 ± 0.001	0.24
Trabecular number (mm^−1^)	1.90 ± 0.03	1.92 ± 0.03	1.93 ± 0.03	1.90 ± 0.03	0.96
Strength, mean ± SE					
Failure load (N)	1936.6 ± 28.9	1936.6 ± 26.7	1967.9 ± 26.1	2019.2 ± 27.9	**0.04**

*Note*: Bold values are significant at *p* < 0.05. Least squares values are adjusted for age, weight, height, physical activity, smoking.

Abbreviation: Q, quartile; SE, standard error.

* *p* < 0.05 versus Q1.

** *p* < 0.05 versus Q2.

### Bone strength

Higher grip strength in men was associated with greater μFEA‐calculated failure load (*p* trend <0.01), with those in the highest quartile having 11% higher failure load (*p* < 0.01) compared to the lowest quartile (Table 2). Among women (Table 3), mean failure load was higher with greater grip strength (*p* = 0.04). Although women in the highest grip strength quartile had 4% greater mean failure load versus the lowest quartile, the difference was not statistically significant (*p* = 0.22).

Results were similar when excluding participants with type 2 diabetes and when considering diabetes as an additional covariate (Supplemental Tables S1–S4). Of note, when women with diabetes were excluded, the association between grip strength and cortical area fraction was no longer statistically significant.

## Discussion

In our cohort of community‐dwelling older men and women, greater hand grip strength was associated with greater distal radius bone strength, estimated as failure load by μFEA. Grip strength was positively associated with total cross‐sectional area in both men and women, and with cortical area fraction in women, but not with bone density or microarchitecture, indicating that the relation with bone strength may be due primarily to the positive association with overall bone size.

Others have reported positive associations between grip strength and estimated distal radius bone strength in cohorts of older men^(^ ^12^ ^)^ and of men and women combined.^(^ ^13^, ^14^ ^)^ Although these results are consistent with ours, prior studies estimated bone strength as the polar strength‐strain index, calculated based on bone geometric properties and density acquired by pQCT. To our knowledge, ours is the first population‐based study of adults to investigate the relation of grip strength with distal radius bone strength estimated by μFEA using HR‐pQCT images, which is an excellent in vivo surrogate for bone strength based on experimental validation.^(^ ^31^ ^)^

Our findings of a positive relation between grip strength and distal radius total cross‐sectional area in both men and women are congruous with those of previous studies utilizing pQCT, including the Osteoporotic Fractures in Men (MrOS) Study cohort of older men,^(^ ^12^ ^)^ a sample of 129 community‐based older women,^(^ ^9^ ^)^ and men and women, mean age 69 years, participating in the Hertfordshire Cohort Study.^(^ ^13^ ^)^ In contrast, Szulc et al.,^(^ ^15^ ^)^ the only other study to use HR‐pQCT, found no association between grip strength and total bone size among men in the Structure of Aging Men's Bones (STRAMBO) cohort. They did observe positive associations for cortical thickness and cortical area fraction. In a group of Japanese men and women spanning a wide age range and measured via pQCT, Kaji et al.^(^ ^14^ ^)^ reported that grip strength was positively associated with cortical thickness and cortical area.

We are aware of only one previous study to evaluate the association of grip strength with longitudinal changes in HR‐pQCT measures at the distal radius in older adults. In a more recent investigation over 8 years of follow‐up in the STRAMBO cohort, men in the lowest quartile of baseline grip strength had accelerated declines in total vBMD, cortical vBMD, cortical thickness, and cortical area, as well as more rapid increases in trabecular area compared to higher grip strength quartiles.^(^ ^32^ ^)^ Similar to our cross‐sectional findings, this longitudinal study found that grip strength was not associated with changes in measures of trabecular microarchitecture (thickness, number, separation), though the lack of association may be attributed to the limitations of how the endocortical surface is defined in longitudinal HR‐pQCT analyses leading to an incorrect characterization of trabecularization at the endocortical surface and subsequently biased estimates of changes in trabecular indices over time.^(^ ^32^ ^)^ While we did not observe consistent associations with vBMD in our study, and neither total cross‐sectional area nor estimated bone strength were evaluated in the STRAMBO study, our results are in line with the longitudinal findings of a positive relation between grip strength and changes in cortical bone thickness.

Although many of our findings are consistent with previous studies, others have observed associations between grip strength and bone measures that we did not. For example, prior investigations have found positive associations between grip strength and cortical area fraction^(^ ^14^ ^)^ and trabecular number.^(^ ^15^ ^)^ In our cohort of older men and women, grip strength was not associated with any measures of bone microarchitecture (cortical porosity, trabecular thickness, trabecular number). Grip strength has previously been positively associated with vBMD in both older men and women.^(^ ^11^, ^14^, ^15^ ^)^ In contrast, there was no association between grip strength and vBMD among the men in our study. We observed a statistically significant inverse association between vBMD and grip strength among the women. If muscle strength does contribute to larger bone size, this finding may be due to larger bones having similar bone mineral content and subsequent lower vBMD, which was previously demonstrated by in vivo and in vitro studies by Zebaze et al.^(^ ^33^ ^)^ Examination of the distribution of vBMD values across the full range of grip strength did not indicate a consistent inverse relation or reveal any outlier values that may have influenced the test for trend. Furthermore, none of the pairwise comparisons among grip strength quartiles were statistically significant. Thus, the magnitude of the relation between grip strength and vBMD is probably modest and unlikely to have a meaningful impact on overall bone strength.

Although we did not use formal statistical testing to compare characteristics between men and women, we observed higher cortical porosity in men compared to women (4.3% vs. 3.7%). While it may be expected that the postmenopausal women in our cohort would have worse cortical porosity compared to the men, previous studies have shown that men have higher cortical porosity at the radius versus women in samples of adults with ages ranging from young to older adulthood.^(^ ^34^, ^35^ ^)^ Older postmenopausal women do, however, demonstrate accelerated increases in cortical porosity with aging compared to men.^(^ ^36^ ^)^ Nevertheless, mean cortical porosity in our cohort of men and women aged 50 years and older was consistent with that seen in older men and women in the population‐based Hertfordshire Cohort Study.^(^ ^37^ ^)^ Thus, the higher cortical porosity in men observed in our study is not unexpected.

The mechanostat theory posits that mechanical strain applied to bone is a determinant of bone remodeling, with greater strain favoring formation over resorption.^(^ ^38^ ^)^ Muscles are a primary source of mechanical strain on bone due to the forces applied directly to the bone surface at insertion points and forces resulting from muscle activity, stimulating the mechanosensing cells in the bone tissue.^(^ ^39^ ^)^ Our findings of a positive association between grip strength and radius cross‐sectional area in both older men and women suggest a role for greater muscle strength in the maintenance of bone size. The periosteal expansion that occurs with aging, resulting in larger bone diameter and consequent greater strength, is considered a compensatory mechanism to offset structural weakening due to cortical thinning from endosteal resorption.^(^ ^40^ ^)^ Our results suggest that mechanical strain produced by muscles may play a role in periosteal apposition in older adults. Further, although greater grip strength was associated with larger bone size in both men and women, an association with cortical thickness was observed only in men, and the magnitude of the association between grip strength and bone failure load was greater in men. These results suggest that increased muscle loading may be a mechanism for the greater periosteal expansion and maintenance of bone strength that occurs in men compared to women,^(^ ^41^ ^)^ though additional longitudinal studies are needed to confirm how muscle strength may influence bone adaptations with aging.

Our study has some important limitations of note. Although we focused on the direct influence of local muscle strength on bone, there are likely additional systemic endocrine and genetic effects on the bone‐muscle relation for which we did not account.^(^ ^3^ ^)^ Additionally, we did not account for muscle mass, which Szulc et al.^(^ ^15^ ^)^ suggest may have a substantial and unique impact on bone characteristics, independent of muscle strength. The muscles involved in generating grip strength, including extensors (digitorum), flexors (digit minima brevis, pollicis longus, digitorum superficialis, digitorum profundus) and other small intrinsic muscles in the hand (lumbricals, interossei, adductor policis) do not insert directly into the distal radius. These muscles are, however, near those muscles that do (brachioradialis, pronator quadratus, tendon of the supinator longus) and are likely good surrogates for their strength. The physical activity index does not distinguish between activities affecting the upper versus lower extremities, nor does it account for earlier life activities that can impact the loading of bone.^(^ ^42^, ^43^ ^)^ Framingham Offspring participants are predominantly White, thus our findings may have limited generalizability to populations comprising individuals of other race/ethnicity groups. The HR‐pQCT measures were collected an average of nearly 2 years after grip strength and covariates were assessed. Both muscle strength and bone may have changed during this time gap, potentially obscuring the true association. Conversely, because grip strength was measured prior to HR‐pQCT, our study has a prospective aspect of temporality that may provide stronger evidence of association than would a purely cross‐sectional study. Finally, any inferences regarding a direct, physiologic impact of muscle strength on bone in older adults must be made with caution because both muscle strength and bone strength are influenced by the lifelong effects of genetic factors, nutritional factors, lifestyle, and hormones. Despite these limitations, our study is among the first to examine the relation of grip strength with HR‐pQCT measures at the distal radius among both men and women. Furthermore, we were able to estimate bone strength based on μFEA. Although our study was not longitudinal by design, grip strength was assessed prior to collection of HR‐pQCT measures and was thus prospective. The Framingham Offspring cohort includes a large sample of community‐dwelling older men and women who are well‐characterized, allowing for control of important potential confounding variables.

In conclusion, in older men and women, greater hand grip strength is associated with larger bone size and greater bone strength at the distal radius. Our findings suggest that loading by muscles may not affect density or microarchitecture, thus the positive relation between muscle strength and bone strength may be driven primarily by bone size. Although these results provide further evidence of the important role of muscle strength in the prevention of fractures in both men and women, it remains unclear whether maintenance or gains in strength in older adults have a meaningful impact on the prevention of age‐related decline in bone strength.

## Author Contributions

**Robert McLean:** Conceptualization; formal analysis; investigation; methodology; writing‐original draft; writing‐review & editing. **Elizabeth (Lisa) Samelson:** Investigation; methodology; writing‐review & editing. **Amanda Lorbergs:** Methodology; writing‐review & editing. **Kerry Broe:** Data curation; project administration; writing‐review & editing. **Marian Hannan:** Conceptualization; investigation; methodology; writing‐review & editing. **Steven Boyd:** Data curation; investigation; methodology; writing‐review & editing. **Mary Bouxsein:** Conceptualization; data curation; methodology; writing‐review & editing. **Douglas Kiel:** Conceptualization; funding acquisition; investigation; methodology; project administration; writing‐review & editing. *Study design*: Robert R. McLean, Elizabeth J. Samelson, Marian T. Hannan, Mary L. Bouxsein, and Douglas P. Kiel. *Study conduct*: Robert R. McLean, Kerry E. Broe, Mary L. Bouxsein, Steven K. Boyd, and Douglas P. Kiel. *Data analysis*: Robert R. McLean. *Data interpretation*: Robert R. McLean, Elizabeth J. Samelson, Amanda L. Lorbergs, Kerry E. Broe, Marian T. Hannan, Mary L. Bouxsein, Steven K. Boyd, and Douglas P. Kiel. *Drafting manuscript*: Robert R. McLean. *Revising manuscript content*: Robert R. McLean, Elizabeth J. Samelson, Amanda L. Lorbergs, Kerry E. Broe, Marian T. Hannan, Mary L. Bouxsein, Steven K. Boyd, and Douglas P. Kiel. *Approving final version of manuscript*: Robert R. McLean, Elizabeth J. Samelson, Amanda L. Lorbergs, Kerry E. Broe, Marian T. Hannan, Mary L. Bouxsein, Steven K. Boyd, and Douglas P. Kiel. Robert R. McLean takes responsibility for the integrity of the data analysis.

## Conflict of Interest

All authors state that they have no conflicts of interest related to this work. Douglas P. Kiel received grant support from Merck Sharp & Dohme in the form of an investigator‐initiated research grant but retained full independence in use and reporting of data generated from this funding.

### Peer Review

The peer review history for this article is available at https://publons.com/publon/10.1002/jbm4.10485.

## Supporting information


**Supplemental Table S1**. Least squares‐adjusted* mean radius bone parameters (± SE) by quartiles of maximum grip strength among 380 **MEN** in the Framingham Osteoporosis Study, excluding participants with diabetes
**Supplemental Table S2**. Least squares‐adjusted* mean radius bone parameters (± SE) by quartiles of maximum grip strength among 561 **WOMEN** in the Framingham Osteoporosis Study, excluding participants with diabetes
**Supplemental Table S3**. Least squares‐adjusted* mean radius bone parameters (± SE) by quartiles of maximum grip strength among 508 **MEN** in the Framingham Osteoporosis Study, adjusted for diabetes
**Supplemental Table S4**. Least squares‐adjusted* mean radius bone parameters (± SE) by quartiles of maximum grip strength among 651 **WOMEN** in the Framingham Osteoporosis Study, adjusted for diabetes
