# Supplementary material for: Higher Hand Grip Strength Is Associated With Greater Radius Bone Size and Strength in Older Men and Women: The Framingham Osteoporosis Study
Source: JBMR Plus. 2021 Mar 30;5(5):e10485. doi: 10.1002/jbm4.10485 (PMC8101610; doi:10.1002/jbm4.10485)
Supplement: Supplementary file 1 — Supplemental Table S1. Least squares‐adjusted* mean radius bone parameters (± SE) by quartiles of maximum grip strength among 380 MEN in the Framingham Osteoporosis Study, excluding participants with diabetes Supplemental Table S2. Least squares‐adjusted* mean radius bone parameters (± SE) by quartiles of maximum grip strength among 561 WOMEN in the Framingham Osteoporosis Study, excluding participants with diabetes Supplemental Table S3. Least squares‐adjusted* mean radius bone parameters (± SE) by quartiles of maximum grip strength among 508 MEN in the Framingham Osteoporosis Study, adjusted for diabetes Supplemental Table S4. Least squares‐adjusted* mean radius bone parameters (± SE) by quartiles of maximum grip strength among 651 WOMEN in the Framingham Osteoporosis Study, adjusted for diabetes [file JBM4-5-e10485-s001.docx]

Supplemental Tables

| Supplemental Table 1. Least squares-adjusted* mean radius bone parameters (± SE) by quartiles of maximum grip strength among 380 **MEN** in the Framingham Osteoporosis Study, excluding participants with diabetes | | | | | |
| --- | --- | --- | --- | --- | --- |
| Bone Parameter | Grip strength quartile | | | | |
|  | Q1  (N=86) | Q2  (N=97) | Q3  (N=96) | Q4  (N=101) | P-value^†^ |
| **Density** |  |  |  |  |  |
| Total density (mg/cm^3^) | 333 ± 7 | 332 ± 6 | 332 ± 6 | 341 ± 7 | 0.73 |
| Trabecular density (mg/cm^3^) | 187 ± 4 | 186 ± 4 | 186 ± 4 | 192 ± 4 | 0.66 |
| Cortical density (mg/cm^3^) | 955 ± 6 | 952 ± 5 | 954 ± 5 | 964 ± 5 | 0.45 |
| **Size/morphology** |  |  |  |  |  |
| Total area (mm^2^) | 366.7 ± 5.9 | 369.7 ± 5.3 | 382.2 ± 5.3 | 391.5 ± 5.6^a,b^ | **0.02** |
| Cortical area fraction (%) | 20.8 ± 0.6 | 20.7 ± 0.5 | 20.8 ± 0.5 | 21.1 ± 0.5 | 0.97 |
| Cortical thickness (mm) | 0.954 ± 0.022 | 0.961 ± 0.020 | 0.982 ± 0.019 | 1.007 ± 0.021 | 0.33 |
| **Microarchitecture** |  |  |  |  |  |
| Cortical porosity (%) | 4.1 ± 0.2 | 4.2 ± 0.2 | 4.4 ± 0.2 | 4.2 ± 0.2 | 0.56 |
| Trabecular thickness (mm) | 0.070 ± 0.001 | 0.070 ± 0.001 | 0.068 ± 0.001 | 0.071 ± 0.001 | 0.58 |
| Trabecular number (mm^-1^) | 2.22 ± 0.03 | 2.21 ± 0.03 | 2.26 ± 0.03 | 2.27 ± 0.03 | 0.34 |
| **Strength** |  |  |  |  |  |
| Failure load (N) | 3132.1 ± 63.0 | 3173.6 ± 55.6 | 3274.3 ± 55.7 | 3482.5 ± 58.4^a,b,c^ | **<0.01** |
| *Adjusted for age, weight, height, physical activity, smoking  ^†^Test for overall difference among means  ^a^P<0.05 vs. Q1  ^b^P<0.05 vs. Q2  ^c^P<0.05 vs. Q3 | | | | | |

| Supplemental Table 2. Least squares-adjusted* mean radius bone parameters (± SE) by quartiles of maximum grip strength among 561 **WOMEN** in the Framingham Osteoporosis Study, excluding participants with diabetes | | | | | |
| --- | --- | --- | --- | --- | --- |
| Bone Parameter | Grip strength quartile | | | | |
|  | Q1  (N=133) | Q2  (N=134) | Q3  (N=144) | Q4  (N=150) | P-value^†^ |
| **Density** |  |  |  |  |  |
| Total density (mg/cm3) | 305 ± 6 | 296 ± 5 | 294 ± 5 | 287 ± 5 | 0.20 |
| Trabecular density (mg/cm3) | 152 ± 4 | 146 ± 3 | 145 ± 3 | 142 ± 3 | 0.28 |
| Cortical density (mg/cm3) | 963 ± 5 | 959 ± 5 | 959 ± 5 | 952 ± 5 | 0.50 |
| **Size/morphology** |  |  |  |  |  |
| Total area (mm2) | 242.1 ± 3.6 | 249.5 ± 3.3 | 253.0 ± 3.2 | 266.2 ± 3.4^a,b,c^ | **<0.01** |
| Cortical area fraction (%) | 20.9 ± 0.5 | 20.3 ± 0.4 | 20.3 ± 0.4 | 19.8 ± 0.4 | 0.44 |
| Cortical thickness (mm) | 0.809 ± 0.016 | 0.795 ± 0.015 | 0.797 ± 0.014 | 0.789 ± 0.015 | 0.85 |
| **Microarchitecture** |  |  |  |  |  |
| Cortical porosity (%) | 3.7 ± 0.2 | 3.7 ± 0.1 | 3.7 ± 0.1 | 3.8 ± 0.1 | 0.97 |
| Trabecular thickness (mm) | 0.066 ± 0.001 | 0.065 ± 0.001 | 0.063 ± 0.001 | 0.064 ± 0.001 | 0.35 |
| Trabecular number (mm-1) | 1.93 ± 0.04 | 1.90 ± 0.03 | 1.91 ± 0.03 | 1.88 ± 0.03 | 0.81 |
| **Strength** |  |  |  |  |  |
| Failure load (N) | 1947.0 ± 31.9 | 1943.9 ± 29.2 | 1954.3 ± 28.2 | 2018.9 ± 29.4 | 0.26 |
| *Adjusted for age, weight, height, physical activity, smoking  ^†^Test for overall difference among means  ^a^P<0.05 vs. Q1  ^b^P<0.05 vs. Q2  ^c^P<0.05 vs. Q3 | | | | | |

| Supplemental Table 3. Least squares-adjusted* mean radius bone parameters (± SE) by quartiles of maximum grip strength among 508 **MEN** in the Framingham Osteoporosis Study, adjusted for diabetes | | | | | |
| --- | --- | --- | --- | --- | --- |
| Bone Parameter | Grip strength quartile | | | | |
|  | Q1  (N=123) | Q2  (N=139) | Q3  (N=120) | Q4  (N=124) | P-value^†^ |
| **Density** |  |  |  |  |  |
| Total density (mg/cm^3^) | 330 ± 6 | 329 ± 5 | 335 ± 6 | 339 ± 6 | 0.63 |
| Trabecular density (mg/cm^3^) | 185 ± 3 | 185 ± 3 | 186 ± 3 | 190 ± 4 | 0.78 |
| Cortical density (mg/cm^3^) | 951 ± 5 | 950 ± 4 | 953 ± 5 | 960 ± 5 | 0.48 |
| **Size/morphology** |  |  |  |  |  |
| Total area (mm^2^) | 367.6 ± 5.1 | 371.3 ± 4.6 | 382.0 ± 4.9 | 390.2 ± 5.3^a,b^ | **0.02** |
| Cortical area fraction (%) | 20.6 ± 0.5 | 20.4 ± 0.4 | 21.0 ± 0.5 | 21.0 ± 0.5 | 0.83 |
| Cortical thickness (mm) | 0.950 ± 0.019 | 0.948 ± 0.017 | 0.985 ± 0.018 | 1.000 ± 0.019 | 0.16 |
| **Microarchitecture** |  |  |  |  |  |
| Cortical porosity (%) | 4.2 ± 0.2 | 4.2 ± 0.1 | 4.4 ± 0.1 | 4.2 ± 0.2 | 0.78 |
| Trabecular thickness (mm) | 0.070 ± 0.001 | 0.070 ± 0.001 | 0.069 ± 0.001 | 0.070 ± 0.001 | 0.87 |
| Trabecular number (mm^-1^) | 2.20 ± 0.02 | 2.21 ± 0.02 | 2.26 ± 0.02 | 2.26 ± 0.03 | 0.23 |
| **Strength** |  |  |  |  |  |
| Failure load (N) | 3102.2 ± 51.8 | 3150.0 ± 45.6 | 3298.6 ± 49.4^a,b^ | 3428.3 ± 52.5^a,b^ | **<0.01** |
| *Adjusted for age, weight, height, physical activity, smoking, diabetes  ^†^Test for overall difference among means  ^a^P<0.05 vs. Q1  ^b^P<0.05 vs. Q2 | | | | | |

| Supplemental Table 4. Least squares-adjusted* mean radius bone parameters (± SE) by quartiles of maximum grip strength among 651 **WOMEN** in the Framingham Osteoporosis Study, adjusted for diabetes | | | | | |
| --- | --- | --- | --- | --- | --- |
| Bone Parameter | Grip strength quartile | | | | |
|  | Q1  (N=160) | Q2  (N=159) | Q3  (N=168) | Q4  (N=164) | P-value^†^ |
| **Density** |  |  |  |  |  |
| Total density (mg/cm3) | 304 ± 5 | 297 ± 5 | 294 ± 5 | 286 ± 5 | 0.14 |
| Trabecular density (mg/cm3) | 149 ± 3 | 147 ± 3 | 147 ± 3 | 144 ± 3 | 0.76 |
| Cortical density (mg/cm3) | 963 ± 5 | 958 ± 4 | 956 ± 4 | 950 ± 5 | 0.34 |
| **Size/morphology** |  |  |  |  |  |
| Total area (mm2) | 241.5 ± 3.3 | 248.3 ± 3.0 | 254.7 ± 3.0^a^ | 267.1 ± 3.2^a,b,c^ | **<0.01** |
| Cortical area fraction (%) | 21.1 ± 0.4 | 20.4 ± 0.4 | 20.2 ± 0.4 | 19.7 ± 0.4 | 0.13 |
| Cortical thickness (mm) | 0.813 ± 0.015 | 0.796 ± 0.014 | 0.793 ± 0.013 | 0.784 ± 0.014 | 0.59 |
| **Microarchitecture** |  |  |  |  |  |
| Cortical porosity (%) | 3.6 ± 0.1 | 3.8 ± 0.1 | 3.7 ± 0.1 | 3.8 ± 0.1 | 0.89 |
| Trabecular thickness (mm) | 0.065 ± 0.001 | 0.064 ± 0.001 | 0.064 ± 0.001 | 0.063 ± 0.001 | 0.61 |
| Trabecular number (mm-1) | 1.91 ± 0.03 | 1.92 ± 0.03 | 1.93 ± 0.03 | 1.90 ± 0.03 | 0.89 |
| **Strength** |  |  |  |  |  |
| Failure load (N) | 1937.2 ± 28.9 | 1937.6 ± 26.7 | 1968.6 ± 26.1 | 2016.9 ± 27.9 | 0.18 |
| *Adjusted for age, weight, height, physical activity, smoking, diabetes  ^†^Test for overall difference among means  ^a^P<0.05 vs. Q1  ^b^P<0.05 vs. Q2  ^c^P<0.05 vs. Q3 | | | | | |
